# Supplementary material for: Principal Component Analysis of Alternative Splicing Profiles Revealed by Long-Read ONT Sequencing in Human Liver Tissue and Hepatocyte-Derived HepG2 and Huh7 Cell Lines
Source: Int J Mol Sci. 2023 Oct 24;24(21):15502. doi: 10.3390/ijms242115502 (PMC10648607; doi:10.3390/ijms242115502)
Supplement: Supplementary file 1 [file ijms-24-15502-s001.zip › Figures S-1 - S-5 legends.pdf]

Figure legends

Figure S-1. The distributions of mean values of transcript isoforms abundance, obtained by averaging the number of transcript isoforms over all biospecimens of a given type. The blue, orange, and grey color correspond to human liver tissue, HepG2 and Huh7 cells, respectively.

Figure S-2. The scores/scores plots for randomly chosen subsets of genes. The subsets of genes were selected from sets of genes used in PCA for Figure 2*c* (panel *a*) and Figure 2*f* (panel *b*).

Figure S-3. Box plots of loadings. Values of loadings were taken from Table S-5.

Figure S-4. The heatmap of distributions of the number transcript isoforms by biospecimens. The color indicates DS values for a gene at the TPM threshold of 10 and varies from blue to red that correspondence to 1 to 6 transcript isoforms per gene.

Figure S-5. The heatmap of 109 transcript isoforms of 31 genes detected in human liver, HepG2 and Huh7 at the TPM threshold of 10 (selected from Table S-7).
