# Supplementary figures and images for: Principal Component Analysis of Alternative Splicing Profiles Revealed by Long-Read ONT Sequencing in Human Liver Tissue and Hepatocyte-Derived HepG2 and Huh7 Cell Lines
Source: Int J Mol Sci. 2023 Oct 24;24(21):15502. doi: 10.3390/ijms242115502 (PMC10648607; doi:10.3390/ijms242115502)

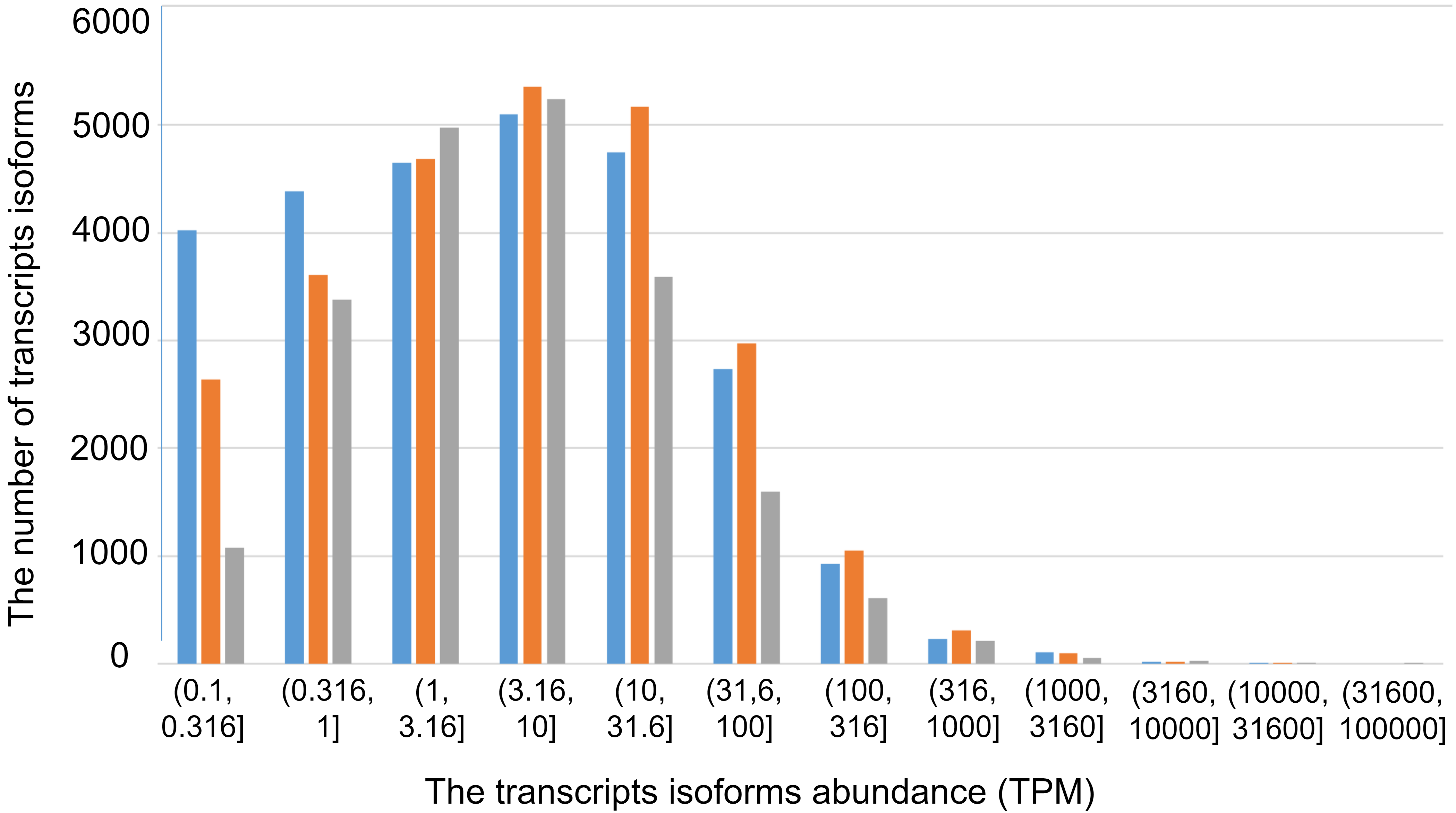

Supplement: Supplementary file 1 [file ijms-24-15502-s001.zip › Figure S-1.tif]

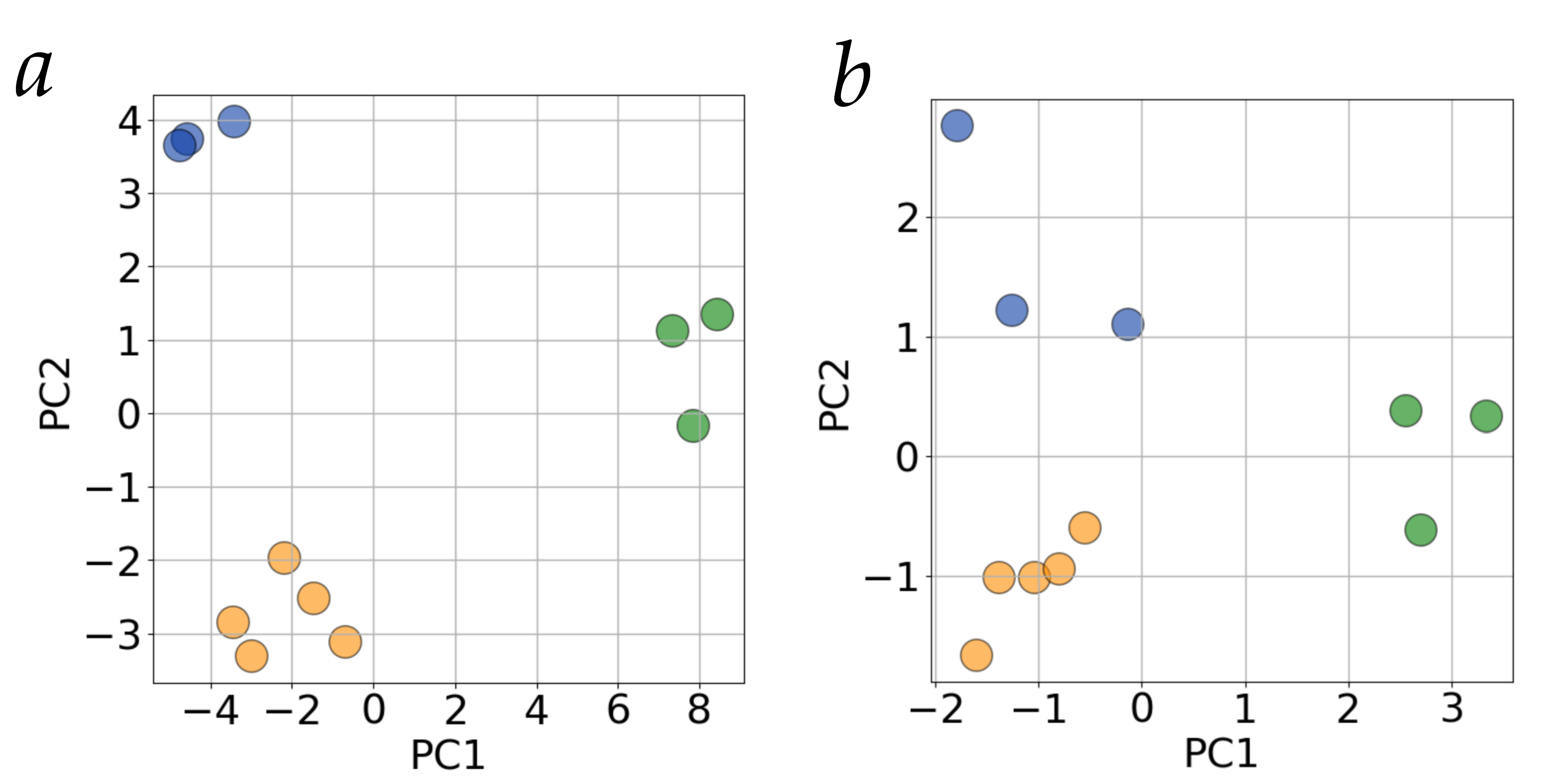

Supplement: Supplementary file 1 [file ijms-24-15502-s001.zip › Figure S-2.tif]

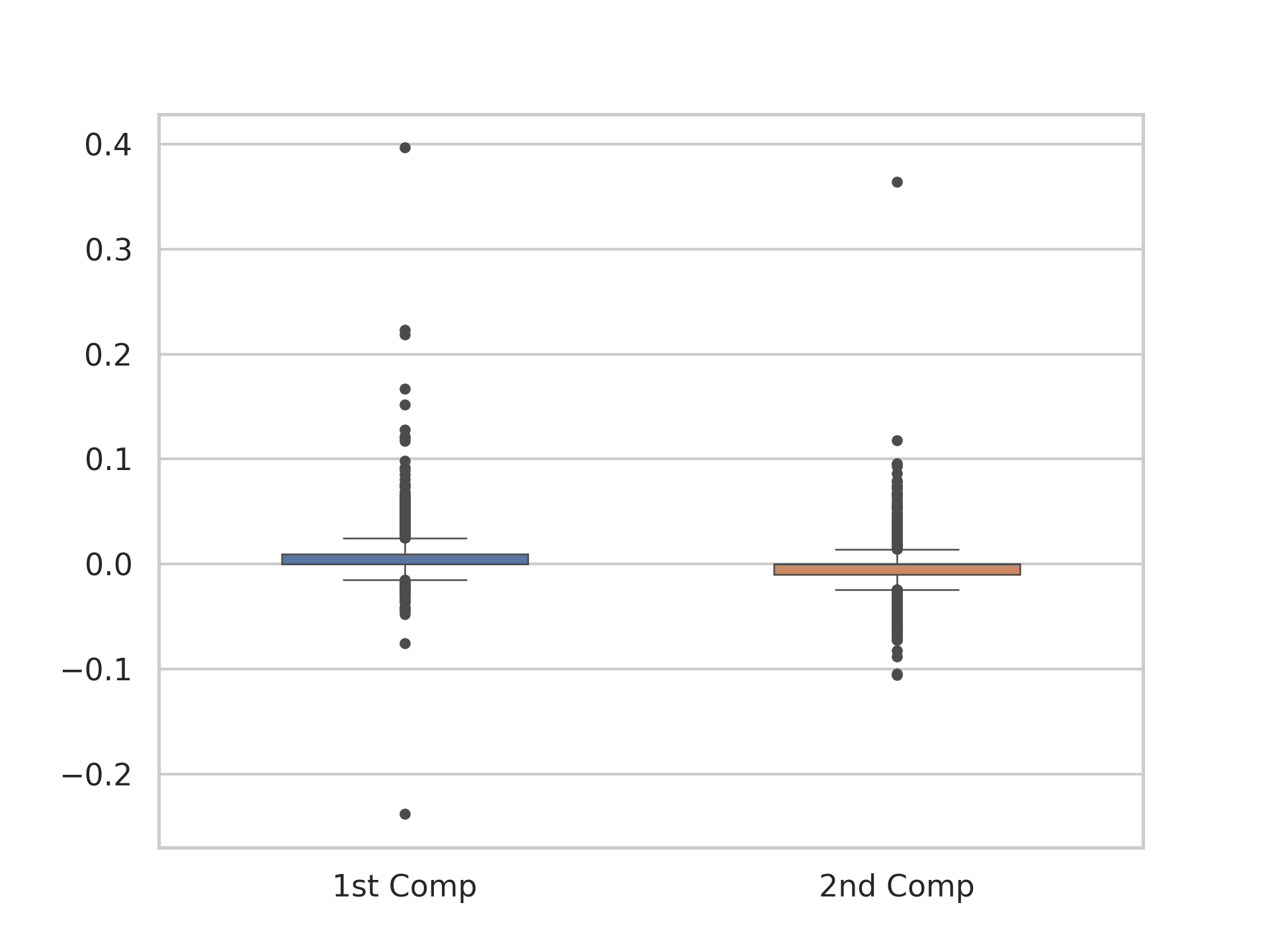

Supplement: Supplementary file 1 [file ijms-24-15502-s001.zip › Figure S-3.tif]

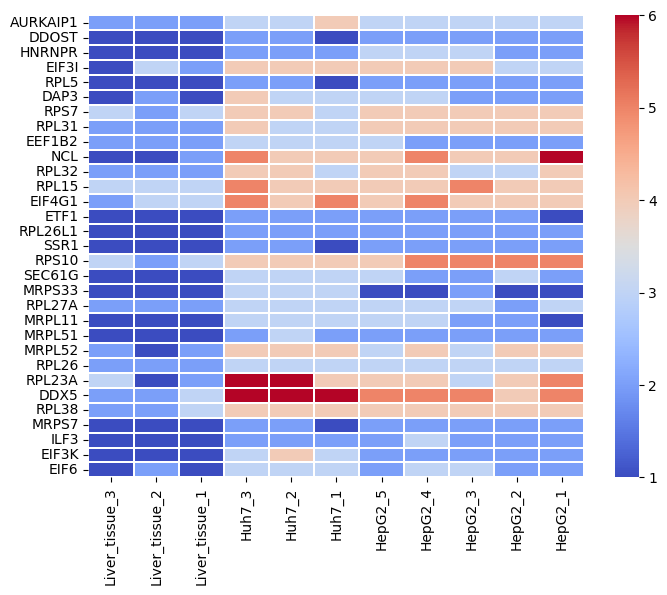

Supplement: Supplementary file 1 [file ijms-24-15502-s001.zip › Figure S-4.tif]

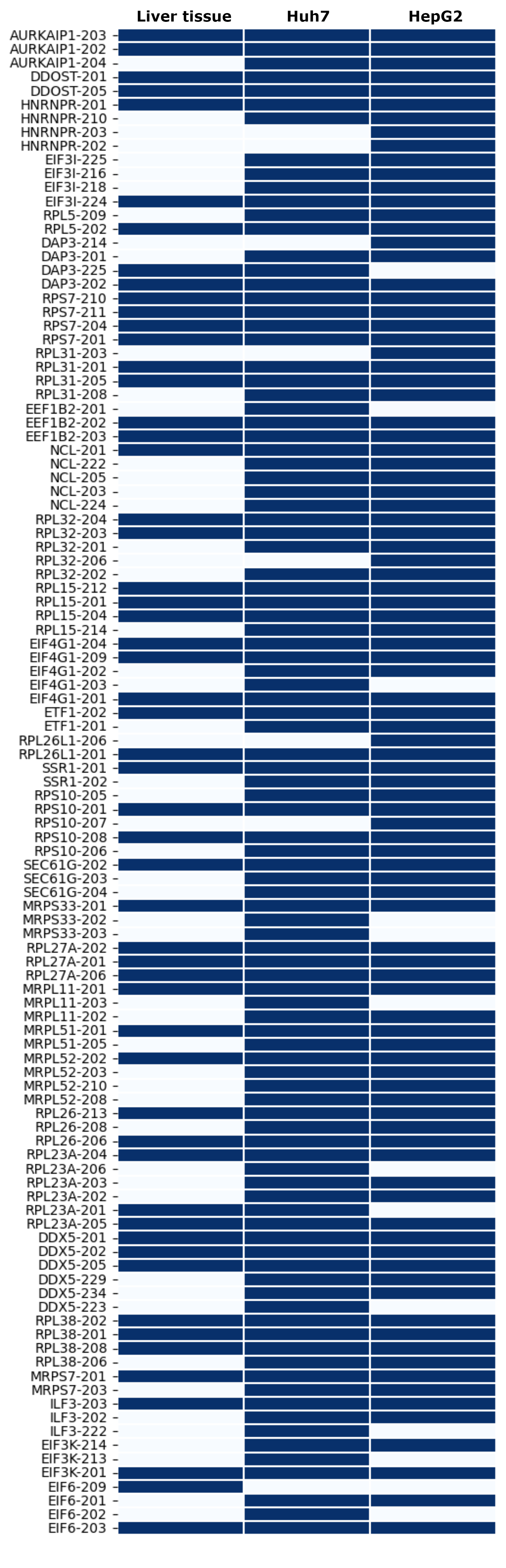

Supplement: Supplementary file 1 [file ijms-24-15502-s001.zip › Figure S-5.tif]
